# Supplementary material for: Ribonucleotide reductases recapitulate biogeographic patterns within virioplankton according to ocean biogeochemistry
Source: bioRxiv. 2026 Feb 24:2026.02.11.705308. Originally published 2026 Feb 12. Preprint. [Version 2] doi: 10.64898/2026.02.11.705308 (PMC12918982; doi:10.64898/2026.02.11.705308)
Supplement: Supplement 1 [file media-1.pdf]

Supplementary Materials

Supplementary Figures 1-7
Supplementary Tables 1-3
Supplementary Methods

Supplementary Figures

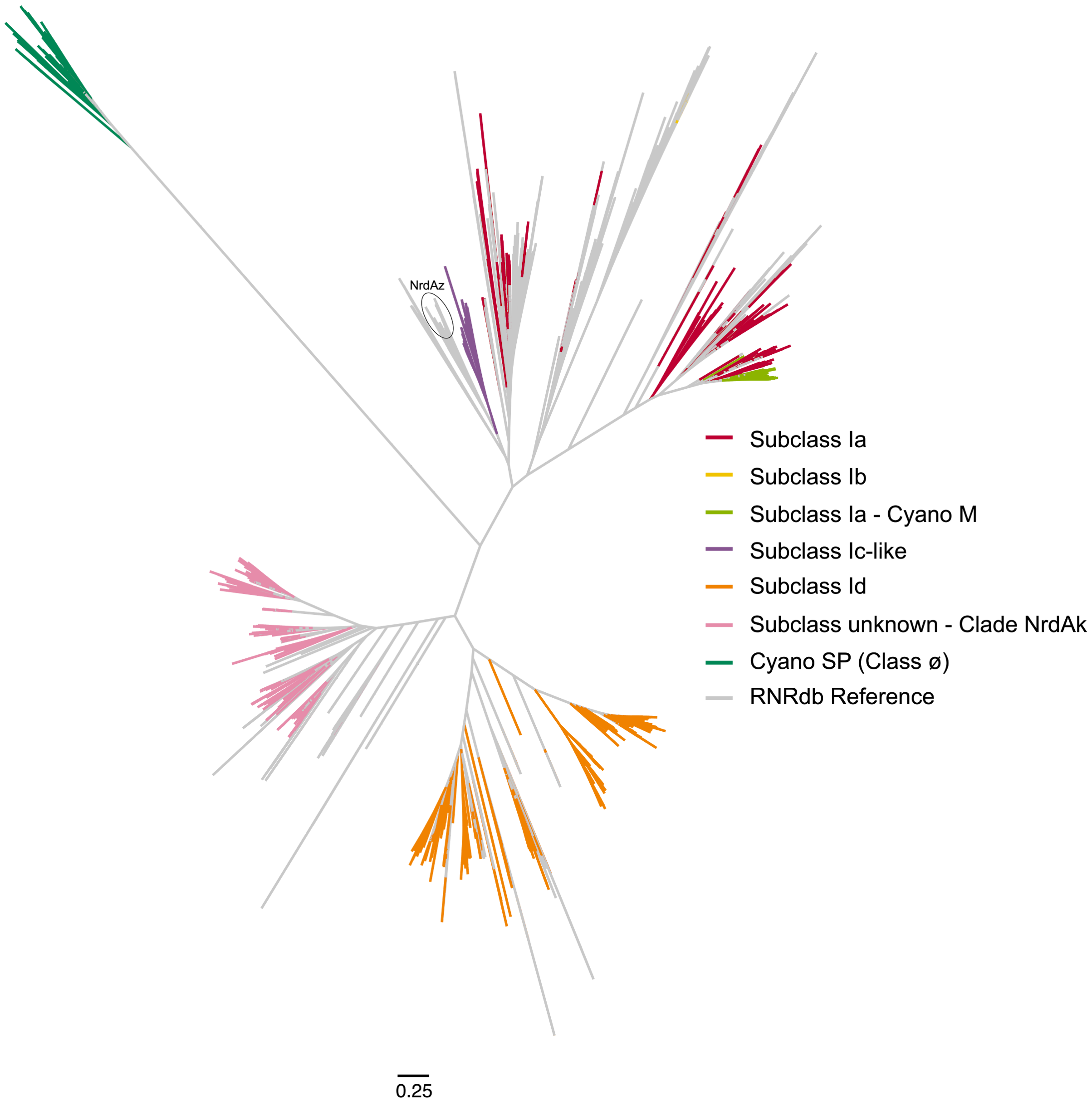

Supplementary Figure S1. RNR Class I and Cyano SP alpha tree used for classification. Colored branches indicate Global Ocean Virome 2.0 sequences.

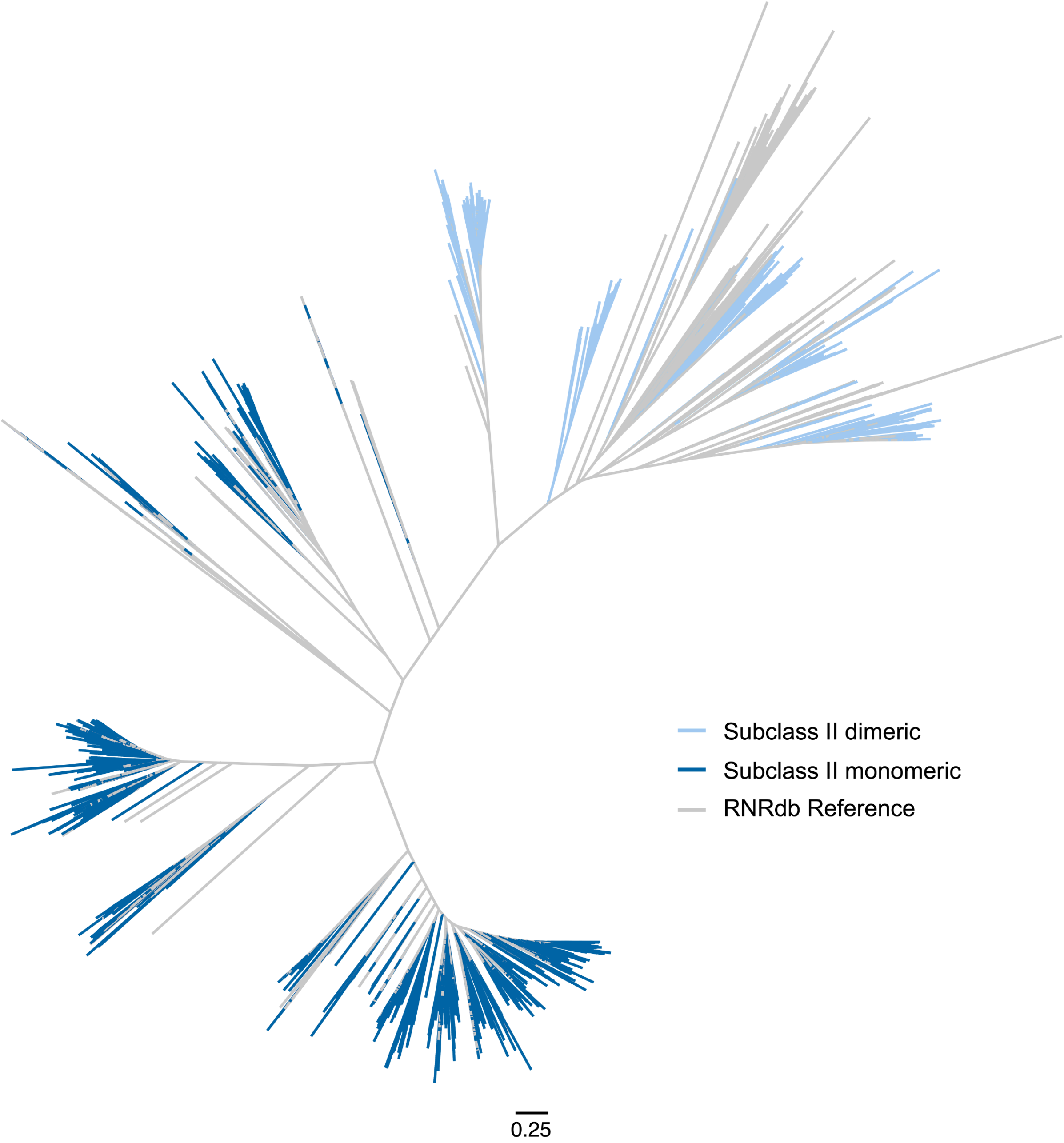

Supplementary Figure S2. Class II tree used for classification.

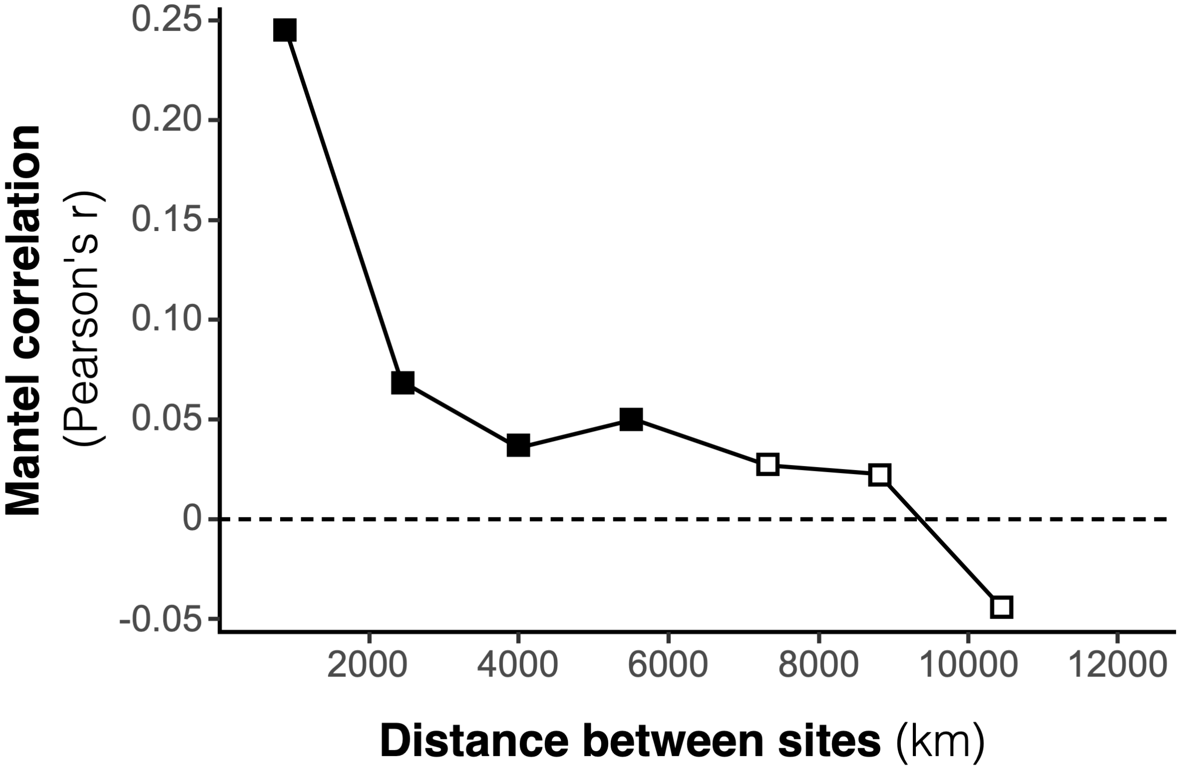

Supplementary Figure S3. Mantel correlogram showing Pearson's correlation between the pairwise Aitchison distance of samples and the geographical distance between samples within seven distance classes. Geographical distance between samples was calculated as the least-cost distance between samples. The Mantel correlogram was constructed using the function `mantel.correlog` from the R package `vegan`. The number of distance classes was calculated using the Sturges equation. Distance classes were tested for significant correlation using permutation Mantel tests performed by the function `mantel` from `vegan`. The Holm method was employed to correct for multiple testing.

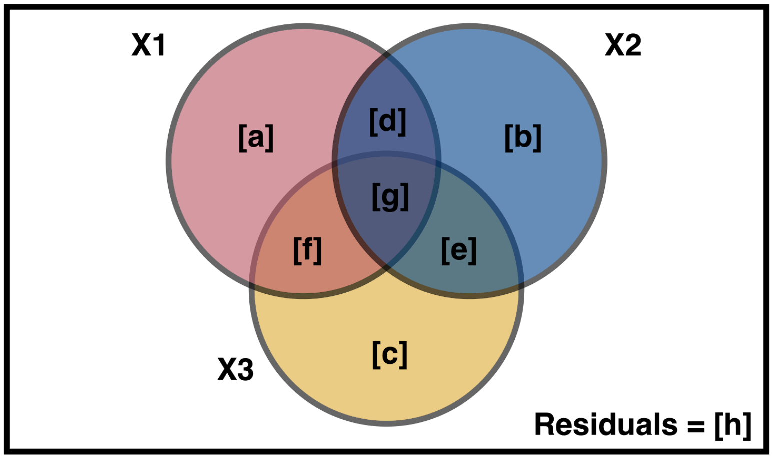

Testable fractions:	Formula:
[a] + [b] + [c] + [d] + [e] + [f] + [g]	Y ~ X1 + X2 + X3
[a] + [b] + [d] + [e] + [f] + [g]	Y ~ X1 + X2 | X3
[a] + [c] + [d] + [e] + [f] + [g]	Y ~ X1 + X3 | X2
[b] + [c] + [d] + [e] + [f] + [g]	Y ~ X2 + X3 | X1
[a] + [d] + [f] + [g]	Y ~ X1
[b] + [d] + [e] + [g]	Y ~ X2
[c] + [e] + [f] + [g]	Y ~ X3
[a] + [d]	Y ~ X1 | X3
[a] + [f]	Y ~ X1 | X2
[b] + [d]	Y ~ X2 | X3
[b] + [e]	Y ~ X2 | X1
[c] + [e]	Y ~ X3 | X1
[c] + [f]	Y ~ X3 | X2
[a]	Y ~ X1 | X2 + X3
[b]	Y ~ X2 | X1 + X3
[c]	Y ~ X3 | X1 + X2

Supplementary Figure S4. Explanation of variation partitioning testable fractions and formulas for significance testing, where Y is the community abundance matrix and X1, 2, and 3 are tables of explanatory variables. Plus signs (+) indicate where multiple groups of explanatory variables are being tested together. Groups of explanatory variables that are being controlled for are shown to the right of bar characters (|). Fractions [d], [e], [f], [g], and [h] are not testable by themselves.

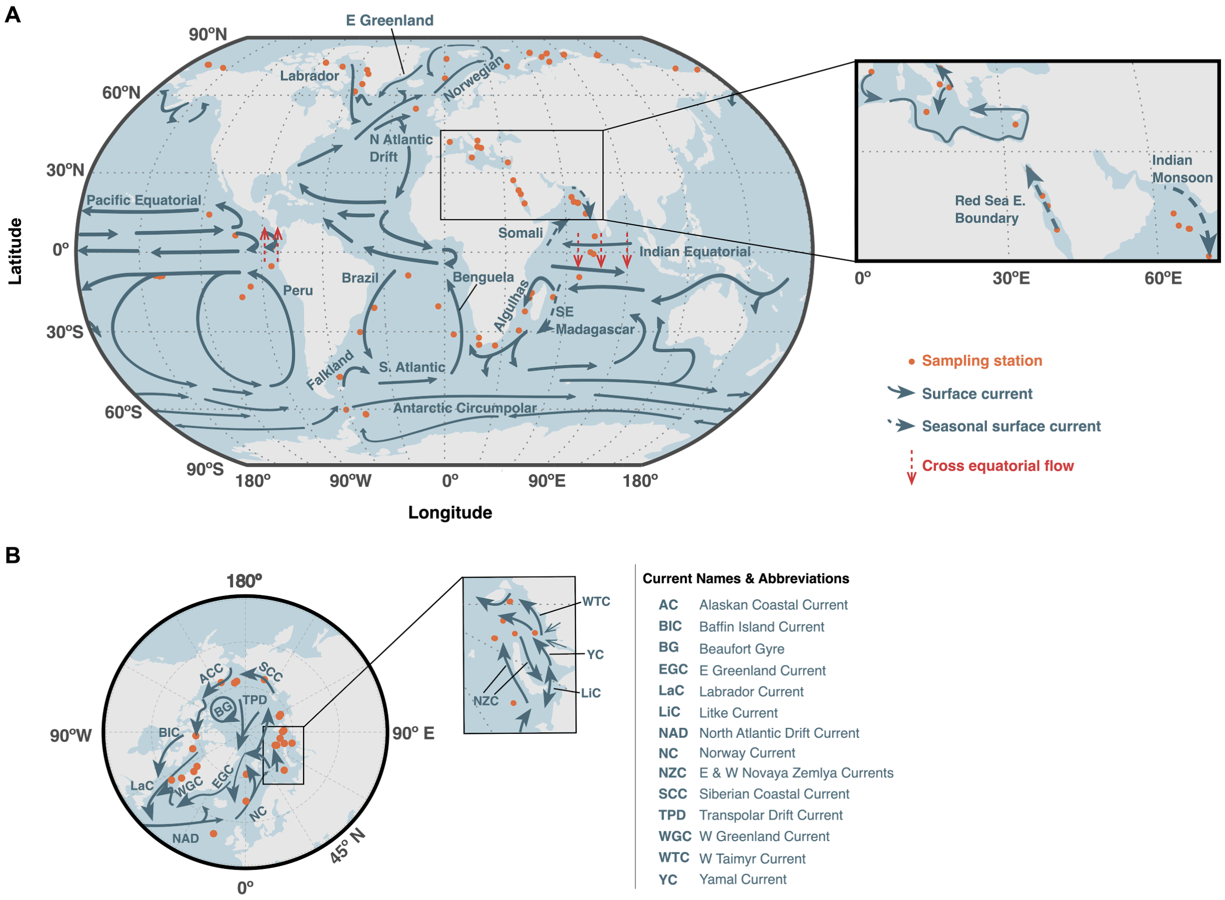

Supplementary Figure S5. Nearby major surface ocean currents and A) *Tara* Oceans sampling stations or B) *Tara* Oceans Polar Circle sampling stations. Currents that alter course seasonally are shown in the orientation expected based on sampling date.

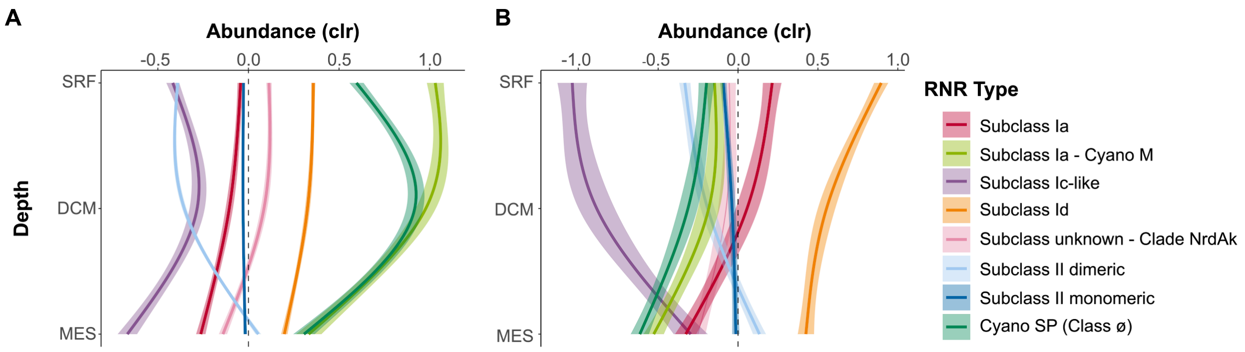

Supplementary Figure S6. General additive models with 95% confidence intervals depicting the relationship between centered log ratio-transformed viral population abundance and depth for A) non-Arctic and B) Arctic samples. Viral population abundance is divided by the RNR type carried. Depths are surface (SRF), deep chlorophyll maximum (DCM), and mesopelagic (MES). Mixed layer depth was excluded because of the relatively few number of samples (n = 5). The bathypelagic depth does not appear because no bathypelagic samples were taken in Arctic waters.

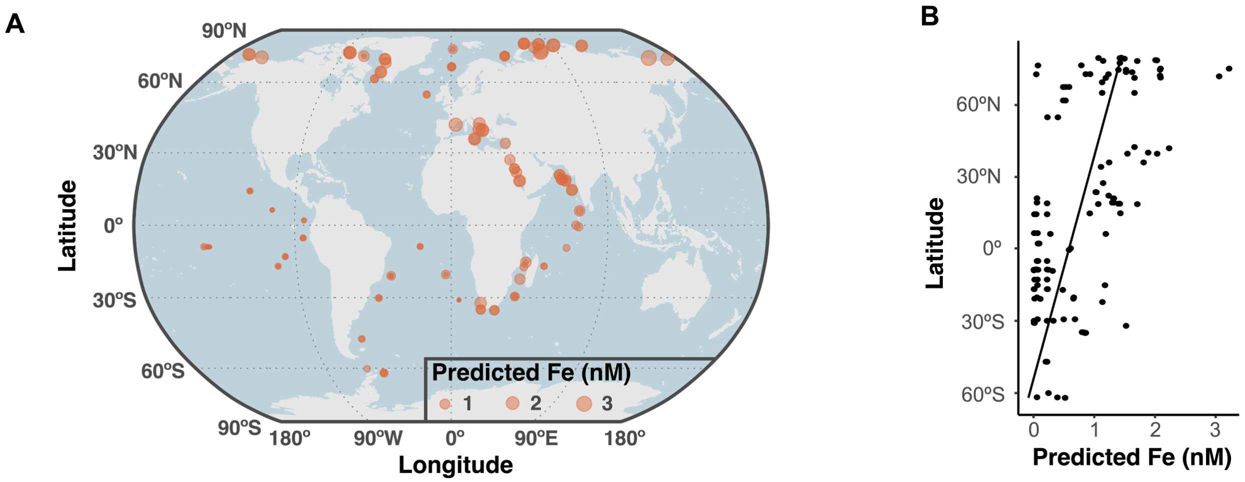

Supplementary Figure S7. A) Predicted iron concentrations (Caputi et al. 2019) for each *Tara* Oceans sample. B) Scatter plot of predicted iron in response to latitude with a line of best fit.

Supplementary Tables

Supplementary Table S1. Conserved residues from Class I RNR alpha subunit and Class II used to validate putative sequences from GOV 2.0 contigs and their position in *E. coli*.

Residue	Position in *E. coli*	Function	Citation
N	437	hydrogen bonds	Kasrayan et al. 2002
C	439	thiyl radical - abstracts H	Mao et al. 1992a; Mao et al. 1992b
E	441	hydrogen bonds	Persson et al. 1997
C	462	active site disulfide bridge	Mao et al. 1992a; Mao et al. 1992b
P	621	active site	Eklund et al. 2001

Supplementary Table S2. Conserved residues from Class III RNR large subunit used to validate putative sequences from GOV 2.0 contigs and their position in Bacteriophage T4.

Residue	Position in T4	Function	Citation
C	79	transient thiyl radical	Andersson et al. 2000
C	290	transient thiyl radical - abstracts H	Olcott et al. 1998; Andersson et al. 2000
C	543	Zn center	Andersson et al. 2000; Logan et al. 2003; Luttringer et al. 2009
C	546	Zn center	Andersson et al. 2000; Logan et al. 2003; Luttringer et al. 2009
C	561	Zn center	Andersson et al. 2000; Logan et al. 2003; Luttringer et al. 2009
C	564	Zn center	Andersson et al. 2000; Logan et al. 2003; Luttringer et al. 2009
G	580	glycyl radical	Logan et al. 1999

Supplementary Table S3. Sources for addition and manual adjustment of ocean currents in Supplementary Figure S5.

Current	Adjustment	Citation
Alaskan Coastal	Addition	Stabeno et al. 2018
Baffin Island	Addition	Curry et al. 2014
Beaufort Gyre	Addition	Cook, n.d.
E &W Novaya Zemlya	Addition	Galimov et al. 2006
Indian Monsoon	Addition	Yi et al. 2018; Schott et al. 2009
Litke	Addition	Panteleev et al. 2007
Mediterranean currents (all)	Addition	Pascual et al. 2017
Red Sea E. Boundary	Addition	Taqi et al. 2019
Siberian Coastal	Addition	Anderson et al. 2011
Somali	Seasonal reversal	Wang et al. 2018
Transpolar Drift	Addition	Constantin and Johnson 2024
Western Taimyr	Addition	Panteleev et al. 2007
Yamal	Addition	Galimov et al. 2006

Supplementary Methods

GOV 2.0 Dataset

The Global Oceans Viromes (GOV) 2.0 dataset is a collection of 145 viral shotgun metagenomes taken during three expeditions (Figure 1): the Malaspina 2010 Circumnavigation (Duarte 2015), *Tara* Oceans, and *Tara* Oceans Polar Circle (TOPC) (Pesant et al. 2015). GOV 2.0 is an expansion of the original GOV dataset (Roux et al. 2016), adding the TOPC samples and additional mesopelagic samples from *Tara* Oceans.

Detailed accounts of sample collection and sequencing can be found in the publications for each of the expeditions. Briefly, Malaspina consists of 14 samples collected between April and June of 2011. The 13 bathypelagic samples were collected from 4,000 m except for two locations where ocean depth was limiting. One sample was taken from the mesopelagic (294 m) to target an oxygen minimum zone (OMZ).

The *Tara* Oceans expedition collected 90 viral samples from 45 stations between October 2009 and December 2011. The TOPC expedition collected 41 samples from 20 stations between June and December of 2013. *Tara* Oceans and TOPC samples were taken from three depths: surface water, the deep chlorophyll maximum (DCM), and the mesopelagic. All surface samples were collected at 5 m depth. The nominal depth of DCM and mesopelagic samples varies by location, as the features were identified at each site using a Rosette Vertical Sampling System (RVSS). DCM samples range from 17 to 177 m depth and mesopelagic samples range from 250 to 1,000 m depth. When no DCM was detected (stations 123, 124, and 125), samples were instead collected at the bottom of the mixed layer, which ranged from 100 to 150 m depth. Additional samples were collected when interesting environmental features were encountered, such as OMZs. All such samples were taken from the mesopelagic.

Processing and storage of samples after collection was highly similar for Malaspina, *Tara* Oceans, and TOPC samples. Following previous filtration steps, seawater (80 L, Malaspina; 20L, *Tara* and TOPC) was filtered through 0.22 μm filters. Viruses were concentrated using the iron chloride flocculation method (John et al. 2011) and viral concentrates were treated with DNase I to remove free DNA. DNA was extracted from viral concentrates using phenol/chloroform (Hurwitz et al. 2013) and sequenced on Illumina HiSeq 2000; Malaspina

viromes were sequenced using 150 bp paired end protocol and *Tara* and TOPC viromes were sequenced using 101 bp paired end protocol.

Specifics of GOV 2.0 sequencing data analysis can be found in the GOV 2.0 publication (Gregory et al. 2019). Briefly, sequences underwent quality control (e.g., primer removal) and were assembled by sample of origin using metaSPAdes (Nurk et al. 2017). Assembled contigs ≥1.5 kb in length were input to VirSorter (Roux et al. 2015) and VirFinder (Ren et al. 2017) to further confirm that contigs were of viral origin. Contigs mapping to the human, cat, or dog genomes were removed from analysis. Only contigs that were verified as viral by VirSorter or VirFinder, had <40% of their length classified as cellular by the contig annotation tool (CAT) (von Meijenfeldt et al. 2019), and were ≥5 kb and linear or ≥1.5 kb and circular were used in further analysis. Remaining contigs were clustered at 95% average nucleotide identity over 80% of their length using nucmer (Kurtz et al. 2004) to create viral populations. In total, 488,130 viral populations were identified in GOV 2.0. Raw virome reads were mapped to viral populations and contigs with <5 kb coverage were removed from further analysis. Average read depth was calculated for the remaining populations and used as a proxy for abundance.

Spatial data Modeling

Testing

Malaspina samples were not included, as they were not used for statistical testing between RNR-containing viral populations and physicochemical data.

A multivariate Mantel correlogram was used to test for spatial autocorrelation in the RNR-containing viral populations (Borcard and Legendre 2012). The correlogram was calculated using the `mantel.correlog` from R package vegan v2.6-4 (Oksanen et al. 2007). Pairwise sample distances were least-cost distances computed in R using the package raster v3.4-5 (Hijmans 2023). Land was masked so that sample distance could only be calculated via ocean routes. A Moran's I test (Moran 1950) was performed for each physicochemical parameter using the function `moran.test` in R package ape v5.4-1 (Paradis and Schliep 2019) to test for significant spatial autocorrelation.

Spatial models

Because both the RNR-containing viral populations and physicochemical variables were significantly autocorrelated, a spatial weight matrix was constructed to control for the autocorrelation.

First, a binary connectivity matrix was manually constructed. A value of 0 or 1 was assigned to each sample pair, with 1 indicating a connection between samples. The matrix was asymmetrical to account for the directionality of ocean currents (i.e. many samples were only connected in a single direction). Samples were connected based on a few simple rules: 1) For each site pair, water was presumed to flow unidirectionally along the major currents (Supplementary Figure S5). For seasonal currents that can change direction, the time of year that sampling took place was considered in assigning directionality. 2) At some distance, sites are no longer connected. The distance limit was set as the first non-significant distance class in the Mantel correlogram (7,255,400 m; Supplementary Figure S3). 3) At the same station, water is well-mixed (in both directions) between the surface and DCM. 4) At the same station, wWater is allowed to pass from the surface and DCM to the mesopelagic, but water may not move up from the mesopelagic unless in an active upwelling zone. Sampling stations located in active upwelling zones were Station 102 - Peru upwelling zone (Boening et al. 10 2012), Station 67 - Benguela upwelling zone (Gordon et al. 1992), Station 82 - Patagonian shelf upwelling (Valla and Piola 2015), and Stations 84 and 85 - Antarctic upwelling (Baker et al. 2023; Tamsitt et al. 2017). 5) Viruses from mesopelagic samples could only be transported to other mesopelagic samples, unless in an upwelling zone. 6) Consider equatorial Ekman drift for samples separated by the equator.

The binary connectivity and least-cost distance matrices were then imported into R and the Hadamard product taken using package matrixcalc v1.0-3 (Novomestky 2022). This resulted in a matrix with least-cost distances for connected samples and zeros for unconnected samples. Based on the shape of the Mantel correlogram (Supplementary Figure S3), a spatial weight matrix was constructed by taking the inverse distance so that closer samples would be more heavily weighted than distant samples.

Because the mixed layer is a real, but leaky, barrier between surface and mesopelagic waters, a weight was used which limited the amount of water allowed to pass from surface and DCM samples to mesopelagic samples. There is no easy answer as to how much water crosses this

barrier in an “average” ocean system, so we tested three additional spatial weight matrices, each with a different barrier of passage from the surface to the mesopelagic. The amount of water permitted to pass through the mixed layer was set at 50%, 10%, and 5% in each additional matrix. These three matrices, in addition to the matrix with no modeled mixed layer boundary, were compared using the function `listw.select` from R package adespatial v0.3-20 (Guénard and Legendre 2022).

Significant eigenvectors were then selected from the spatial weight matrix that explained the most variability (highest adjusted R^2 value) in the RNR-containing viral population (based on the centered log ratio-transformed values) for use in statistical analysis. This was done using the adespatial function `mem.select`. The spatial autocorrelation was best explained by the matrix that put no time limits on site connections and allowed 50% of viruses to cross the epi- to mesopelagic mixed layer boundary.

References

Anderson, L. G., G. Björk, S. Jutterström, et al. 2011. “East Siberian Sea, an Arctic Region of Very High Biogeochemical Activity.” *Biogeosciences* 8 (6). https://d-nb.info/1142822095/34.

Andersson, J., M. Westman, M. Sahlin, and B. M. Sjoberg. 2000. “Cysteines Involved in Radical Generation and Catalysis of Class III Anaerobic Ribonucleotide Reductase. A Protein Engineering Study of Bacteriophage T4 NrdD.” *The Journal of Biological Chemistry* 275 (26): 19449–19455. https://doi.org/10.1074/jbc.M001278200.

Baker, L. E., A. Mashayek, and A. C. Naveira Garabato. 2023. “Boundary Upwelling of Antarctic Bottom Water by Topographic Turbulence.” *AGU Advances* 4 (5). https://doi.org/10.1029/2022av000858.

Boening, Carmen, Josh K. Willis, Felix W. Landerer, R. Steven Nerem, and John Fasullo. 10 2012. “The 2011 La Niña: So Strong, the Oceans Fell.” *Geophysical Research Letters* 39: L19602. https://doi.org/10.1029/2012GL053055.

Borcard, Daniel, and Pierre Legendre. 2012. “Is the Mantel Correlogram Powerful Enough to Be Useful in Ecological Analysis? A Simulation Study.” *Ecology* 93 (6): 1473–1481. https://doi.org/10.1890/11-1737.1.

Caputi, Luigi, Quentin Carradec, Damien Eveillard, et al. 2019. “Community‐Level Responses to Iron Availability in Open Ocean Plankton Ecosystems.” *Global Biogeochemical Cycles* 33 (3): 391–419. https://doi.org/10.1029/2018GB006022.

Constantin, A., and R. S. Johnson. 2024. “The Dynamics of the Transpolar Drift Current.” *Geophysical and Astrophysical Fluid Dynamics* 118 (3): 165–182.

https://doi.org/10.1080/03091929.2024.2351919.

Cook, Jack. n.d. “Arctic Ocean Currents.” Woods Hole Oceanographic Institution. Accessed December 30, 2025. https://www.whoi.edu/ocean-learning-hub/multimedia/arctic-currents-mapped/.

Curry, B., C. M. Lee, B. Petrie, R. E. Moritz, and R. Kwok. 2014. “Multiyear Volume, Liquid Freshwater, and Sea Ice Transports through Davis Strait, 2004–10.” *Journal of Physical Oceanography* 44: 1244–1266. https://doi.org/10.1175/JPO-D-13-0177.1.

Duarte, Carlos M. 2015. “Seafaring in the 21St Century: The Malaspina 2010 Circumnavigation Expedition.” In *Limnology and Oceanography Bulletin*, vol. 24, 24. no. 1. Preprint. https://doi.org/10.1002/lob.10008.

Eklund, H., U. Uhlin, M. Färnegårdh, D. T. Logan, and P. Nordlund. 2001. “Structure and Function of the Radical Enzyme Ribonucleotide Reductase.” *Progress in Biophysics and Molecular Biology* 77 (3): 177–268. https://doi.org/10.1016/s0079-6107(01)00014-1.

Galimov, E. M., L. A. Kodina, O. V. Stepanets, and G. S. Korobeinik. 2006. “Biogeochemistry of the Russian Arctic. Kara Sea: Research Results under the SIRRO Project, 1995-2003.” *Geochemistry International* 44: 1053–1104. https://doi.org/10.1134/S0016702906110012.

Gordon, Arnold L., Ray F. Weiss, William M. Smethie Jr., and Mark J. Warner. 1992. “Thermocline and Intermediate Water Communication between the South Atlantic and Indian Oceans.” *Journal of Geophysical Research*, Int. Indian Ocean Monogr., vol. 97 (C5): 7223. https://doi.org/10.1029/92JC00485.

Gregory, Ann C., Ahmed A. Zayed, Nádia Conceição-Neto, et al. 2019. “Marine DNA Viral Macro- and Microdiversity from Pole to Pole.” *Cell* 177 (5): 1109–1123.e14. https://doi.org/10.1016/j.cell.2019.03.040.

Guénard, Guillaume, and Pierre Legendre. 2022. “Hierarchical Clustering with Contiguity Constraint in R.” In *Journal of Statistical Software*, vol. 103, 103. no. 7. Preprint. https://doi.org/10.18637/jss.v103.i07.

Hijmans, Robert J. 2023. *Raster: Geographic Data Analysis and Modeling*. https://CRAN.R-project.org/package=raster.

Hurwitz, Bonnie L., Li Deng, Bonnie T. Poulos, and Matthew B. Sullivan. 2013. “Evaluation of Methods to Concentrate and Purify Ocean Virus Communities through Comparative, Replicated Metagenomics.” In *Environmental Microbiology*, vol. 15, 15. no. 5. Preprint. https://doi.org/10.1111/j.1462-2920.2012.02836.x.

John, Seth G., Carolina B. Mendez, Li Deng, et al. 2011. “A Simple and Efficient Method for Concentration of Ocean Viruses by Chemical Flocculation.” *Environmental Microbiology Reports* 3 (2): 195–202. https://doi.org/10.1111/j.1758-2229.2010.00208.x.

Kasrayan, Alex, Annika L. Persson, Margareta Sahlin, and Britt-Marie Sjoberg. 2002. “The Conserved Active Site Asparagine in Class I Ribonucleotide Reductase Is Essential for Catalysis.” *The Journal of Biological Chemistry* 277 (8): 5749–5755. https://doi.org/10.1074/jbc.M106538200.

Kurtz, Stefan, Adam Phillippy, Arthur L. Delcher, et al. 2004. “Versatile and Open Software for Comparing Large Genomes.” *Genome Biology* 5 (2): R12. https://doi.org/10.1186/gb-2004-5-2-r12.

Logan, Derek T., Etienne Mulliez, Karl-Magnus Larsson, et al. 2003. “A Metal-Binding Site in the Catalytic Subunit of Anaerobic Ribonucleotide Reductase.” *Proceedings of the National Academy of Sciences of the United States of America* 100 (7): 3826–3831. https://doi.org/10.1073/pnas.0736456100.

Logan, D. T., J. Andersson, B. M. Sjöberg, and P. Nordlund. 1999. “A Glycyl Radical Site in the Crystal Structure of a Class III Ribonucleotide Reductase.” *Science* 283 (5407): 1499–1504. https://doi.org/10.1126/science.283.5407.1499.

Luttringer, Florence, Etienne Mulliez, Bernard Dublet, David Lemaire, and Marc Fontecave. 2009. “The Zn Center of the Anaerobic Ribonucleotide Reductase from E. Coli.” *Journal of Biological Inorganic Chemistry: JBIC* 14 (6): 923–933. https://doi.org/10.1007/s00775-009-0505-9.

Mao SS, Holler TP, Yu GX, Bollinger JM Jr, Booker S, Johnston MI, Stubbe J. 1992a. “A Model for the Role of Multiple Cysteine Residues Involved in Ribonucleotide Reduction: Amazing and Still Confusing.” *Biochemistry* 31 (40): 9733–9743. https://doi.org/10.1021/bi00155a029.

Mao SS, Yu GX, Chalfoun D, Stubbe J. 1992b. “Characterization of C439SR1, a Mutant of Escherichia Coli Ribonucleotide Diphosphate Reductase: Evidence That C439 Is a Residue Essential for Nucleotide Reduction and C439SR1 Is a Protein Possessing Novel Thioredoxin-like Activity.” *Biochemistry* 31 (40): 9752–9759. https://doi.org/10.1021/bi00155a031.

Meijenfeldt, F. A. Bastiaan von, Ksenia Arkhipova, Diego D. Cambuy, Felipe H. Coutinho, and Bas E. Dutilh. 2019. “Robust Taxonomic Classification of Uncharted Microbial Sequences and Bins with CAT and BAT.” *Genome Biology* 20 (1): 217. https://doi.org/10.1186/s13059-019-1817-x.

Moran, P. A. P. 1950. “Notes on Continuous Stochastic Phenomena.” *Biometrika* 37 (1-2): 17–23. https://www.ncbi.nlm.nih.gov/pubmed/15420245.

Novomestky, Frederick. 2022. *Matrixcalc: Collection of Functions for Matrix Calculations*. https://CRAN.R-project.org/package=matrixcalc.

Nurk, Sergey, Dmitry Meleshko, Anton Korobeynikov, and Pavel A. Pevzner. 2017. “metaSPAdes: A New Versatile Metagenomic Assembler.” *Genome Research* 27 (5): 824–834. https://doi.org/10.1101/gr.213959.116.

Oksanen, Jari, Roeland Kindt, Pierre Legendre, et al. 2007. “The Vegan Package.” *Community Ecology Package* 10 (631-637): 719. https://www.researchgate.net/profile/Gavin_Simpson/publication/228339454_The_vegan_Package/links/0912f50be86bc29a7f000000/The-vegan-Package.pdf.

Olcott, M. C., J. Andersson, and B. M. Sjöberg. 1998. “Localization and Characterization of Two Nucleotide-Binding Sites on the Anaerobic Ribonucleotide Reductase from Bacteriophage T4.” *The Journal of Biological Chemistry* 273 (38): 24853–24860.

https://doi.org/10.1074/jbc.273.38.24853.

Panteleev, G., A. Proshutinsky, M. Kulakov, D. A. Nechaev, and W. Maslowski. 2007. “Investigation of the Summer Kara Sea Circulation Employing a Variational Data Assimilation Technique.” *Journal of Geophysical Research: Oceans* 112 (C4). https://doi.org/10.1029/2006JC003728.

Paradis, Emmanuel, and Klaus Schliep. 2019. “Ape 5.0: An Environment for Modern Phylogenetics and Evolutionary Analyses in R.” In *Bioinformatics*, vol. 35, 35. Preprint. https://doi.org/10.1093/bioinformatics/bty633.

Pascual, Marta, Borja Rives, Celia Schunter, and Enrique Macpherson. 2017. “Impact of Life History Traits on Gene Flow: A Multispecies Systematic Review across Oceanographic Barriers in the Mediterranean Sea.” *PloS One* 12 (5): e0176419. https://doi.org/10.1371/journal.pone.0176419.

Persson, Annika L., Mathias Eriksson, Bettina Katterle, Stephan Pötsch, Margareta Sahlin, and Britt-Marie Sjöberg. 1997. “A New Mechanism-Based Radical Intermediate in a Mutant R1 Protein Affecting the Catalytically Essential Glu441 inEscherichia Coli Ribonucleotide Reductase.” *The Journal of Biological Chemistry* 272 (50): 31533–31541. https://doi.org/10.1074/jbc.272.50.31533.

Pesant, Stéphane, Tara Oceans Consortium Coordinators, Fabrice Not, et al. 2015. “Open Science Resources for the Discovery and Analysis of Tara Oceans Data.” In *Scientific Data*, vol. 2, 2. no. 1. Preprint. https://doi.org/10.1038/sdata.2015.23.

Ren, Jie, Nathan A. Ahlgren, Yang Young Lu, Jed A. Fuhrman, and Fengzhu Sun. 2017. “VirFinder: A Novel K-Mer Based Tool for Identifying Viral Sequences from Assembled Metagenomic Data.” *Microbiome* 5 (1): 69. https://doi.org/10.1186/s40168-017-0283-5.

Roux, Simon, Jennifer R. Brum, Bas E. Dutilh, et al. 2016. “Ecogenomics and Potential Biogeochemical Impacts of Globally Abundant Ocean Viruses.” *Nature* 537 (7622): 689–693. https://doi.org/10.1038/nature19366.

Roux, Simon, Francois Enault, Bonnie L. Hurwitz, and Matthew B. Sullivan. 2015. “VirSorter: Mining Viral Signal from Microbial Genomic Data.” In *PeerJ*, vol. 3, 3. Preprint. https://doi.org/10.7717/peerj.985.

Schott, Friedrich A., Shang Ping Xie, and Julian P. McCreary. 2009. “Indian Ocean Circulation and Climate Variability.” *Reviews of Geophysics* 47. https://doi.org/10.1029/2007RG000245.

Stabeno, Phyllis, Nancy Kachel, Carol Ladd, and Rebecca Woodgate. 2018. “Flow Patterns in the Eastern Chukchi Sea: 2010–2015.” *Journal of Geophysical Research, C: Oceans* 123: 1177–1195. https://doi.org/10.1002/2017JC013135.

Tamsitt, Veronica, Henri F. Drake, Adele K. Morrison, et al. 2017. “Spiraling Pathways of Global Deep Waters to the Surface of the Southern Ocean.” In *Nature Communications*, vol. 8, 8. no. 1. Preprint. https://doi.org/10.1038/s41467-017-00197-0.

Taqi, Ahmed Mohammed, Abdullah Mohammed Al-Subhi, Mohammed Ali Alsaafani, and Cheriyeri Poyil Abdulla. 2019. “Estimation of Geostrophic Current in the Red Sea Based on

Sea Level Anomalies Derived from Extended Satellite Altimetry Data." *Ocean Science* 15 (3): 477–488. https://doi.org/10.5194/os-15-477-2019.

Valla, Daniel, and Alberto R. Piola. 2015. "Evidence of Upwelling Events at the Northern Patagonian Shelf Break." In *Journal of Geophysical Research: Oceans*, vol. 120, 120. no. 11. Preprint. https://doi.org/10.1002/2015jc011002.

Wang, He, Julie L. McClean, Lynne D. Talley, and Stephen Yeager. 2018. "Seasonal Cycle and Annual Reversal of the Somali Current in an Eddy‐resolving Global Ocean Model." *Journal of Geophysical Research. Oceans* 123 (9): 6562–6580. https://doi.org/10.1029/2018jc013975.

Yi, Xing, Birgit Hünicke, Nele Tim, and Eduardo Zorita. 2018. "The Relationship between Arabian Sea Upwelling and Indian Monsoon Revisited in a High Resolution Ocean Simulation." *Climate Dynamics* 50: 201–213. https://doi.org/10.1007/s00382-017-3599-8.
